# Virgin Polystyrene Microparticles Exposure Leads to Changes in Gills DNA and Physical Condition in the Mediterranean Mussel *Mytilus Galloprovincialis*

**DOI:** 10.3390/ani11082317

**Published:** 2021-08-05

**Authors:** Paula Masiá, Alba Ardura, Eva García-Vázquez

**Affiliations:** Department of Functional Biology, Faculty of Medicine, University of Oviedo, C/Julian Claveria s/n, 33006 Oviedo, Spain; arduraalba@uniovi.es (A.A.); egv@uniovi.es (E.G.-V.)

**Keywords:** condition index, DNA degradation, marine biota, microplastics, marine conservation

## Abstract

**Simple Summary:**

Microplastic pollution is damaging ecosystems and marine organisms worldwide, and, as this problem is becoming greater, the fate of these marine organisms should be studied. In this study, the physical condition and the DNA integrity of gills of Mediterranean mussels (*Mytilus galloprovincialis*) have been studied under four microplastic concentrations for 21 days. A worse physical status was shown at the end of the experiment when exposed to highest concentrations; however, DNA damage was higher when exposed to lower concentrations. These results prove that mussels can be affected by direct exposure even at a low microplastic concentration due to their filter-feeding behavior, making them more vulnerable to this type of pollution.

**Abstract:**

The ever-growing concentration of microplastics in the marine environment is leading to a plethora of questions regarding marine organisms’ present and future health status. In this article, the Mediterranean mussel (*Mytilus galloprovincialis*), a commercial species distributed worldwide, has been exposed to 21 daily doses of polystyrene microparticles (10 µm) at four different concentrations that are environmentally realistic (control: no microplastics, C1: 0.02 mg/L, C2: 0.2 mg/L, and C3: 2 mg/L). The physical status through the condition index, and damages in DNA integrity in gills, through DNA fragmentation, were determined. Results showed a minor effect on DNA integrity but a worse physical status at higher doses. Results could be interpreted as a decrease in mussel feeding activity/filtration rates when exposed to high microplastic concentrations, thus reducing the direct exposure to microplastics in gills. These effects could be happening currently and/or may happen in the near future, threatening populations inhabiting microplastics-polluted environments.

## 1. Introduction

Plastic pollution is one of the most abundant type of pollution worldwide, with estimates of more than 5 trillion plastics particles floating at sea [1], corresponding to ~80% of all marine litter [2]. Most of this plastic litter is constituted by small plastic fragments that come from the degradation of plastics debris, or as a consequence of their direct manufacture, especially for personal care and cosmetic products [3]. Microplastic particles have been reported in a broad range of ecosystems and organisms in the marine environment [4]. Due to currents, winds, and hydrodynamic processes, microplastic can be found in every ecosystem, even with low anthropogenic pressure, such as Antarctica [5], coral reefs [6], and deep marine environments [7]. Due to their ubiquity and small size, microplastics are bioavailable for a great number of marine organisms [8], and therefore reports of ingestion by marine animals are numerous [9], as well as their physical and ecotoxicological implications [10,11,12,13]. Animals reporting ingestion of microplastics range from planktonic species [14,15], corals [16], and cnidarians [17] to fishes [18] and top predators [19]. Filter feeders and pelagic feeders exhibit the highest rates of microplastic consumption [20].

The most abundant types of polymers reported in marine settings are polyethylene (PE), polystyrene (PS), polypropylene (PP), and polyvinyl chloride (PVC) [21], polyethylene being one of the most abundant types in most common plastic products [22], such as plastic bags, stretch film, food packaging, etc. The chemical composition is a key factor for the ability of microplastics to reach different types of marine organisms. For example, plastics exhibiting a high buoyancy, such as low-density polymers, can reach a higher number of marine organisms, especially filter feeders or plankton feeders [23]. On the other hand, high-density polymers are prone to sink, therefore reaching deposit feeders [24]. Different degrees of toxicological risks have been reported depending on the physical properties of microplastics, as polymers with a large and hydrophobic surface area, such as polystyrene, can adsorb a broader range of hazardous compounds found in the marine environment [25,26]. Examples are compounds such as polychlorinated biphenyls (PCBs), organochlorine pesticides, or bisphenol A [25], which have been reported to be carcinogenic, mutagenic, and endocrine disruptors [27]. The ingestion of microplastics could act as a pathway to expose organisms to these hazardous compounds [26]. Once in the organism, microplastics can accumulate or be eliminated via fecal pellets, although the dynamics of the process are still unknown in most cases. Fernandez and Albentosa [28] showed that, in *Mytilus* mussels, 85% of the microplastics were eliminated after 6 days of depuration, having microplastics with >10 μm fastest rates of elimination. Moreover, the egestion of microplastics by fecal pellets in this species can suppose an ecological problem, as microplastics can sink and contaminate the bottom sediments, reaching detritus feeders [29,30] and sediment-dwelling organisms [31]. However, Browne et al. [32] showed that microparticles (3 μm and 9.6 μm) ingested by *Mytilus edulis* could translocate from the gut to the circulatory system within 3 days and be retained for 48 days; therefore, not all the microparticles are egested, since some are stored in mussel tissues, at least for some time.

Many marine organisms have been studied regarding microplastic’s physical and toxicological effects, such as crustaceans, fish, and mollusks [21], *Mytilus* genus being the most studied within the last group. Detrimental effects in terms of ecotoxicological and inflammatory responses have been reported for this genus [33,34]. As an example, effects on energy budget, enzymes, and oxidative responses have been described in *Mytilus coruscus* after two weeks of exposure to polyethylene microspheres [35], as well as an increase in hemocyte mortality and reactive oxygen species (ROS) production in *Mytilus* spp. after 96 h exposure [36]. Détrée and Gallardo-Escárate [37] found changes at transcriptome level after an acute exposure to polystyrene microplastics (24 h) in *Mytilis galloprovincialis*, and Mičić et al. [38] demonstrated that hazardous compounds found in marine environments can cause DNA damage and apoptosis in this species. As microplastics can enhance the accumulation of toxic compounds in organisms’ tissues [33], their combined effect is also being studied. Recently, Han et al. [39] proved synergistic immunotoxic effects in *M. coruscus* when microplastics (500 nm size) were combined with antibiotics after 4 weeks exposure, arriving at the same conclusion as Tang et al. [40], in which the study of immunotoxicity and neurotoxicity effects of bisphenol A were aggravated in *Tegillarca granosa* when combined with microplastics (490 ± 11 nm size) after 2 weeks exposure. Both experiments were carried out at environmentally realistic concentrations (0.26 mg/L and 1 mg/L MPs, respectively), although most of the experiments regarding microplastics exposure use unrealistic doses that are higher than those we can find in the water [20].

Moreover, the effects of microplastics on mussels at a physiological level are reflected in changes in the physical condition of the adults, such as a reduction in byssus production [41], attachment strength [42], or a reduction of the body index [43]; however, changes in the latter are dissimilar, as some authors had not found differences, or marginally significant differences [44,45,46]. For these reasons, it is possible that wild *Mytilus* populations exposed to high plastic pollution are, or will be endangered in the near future. Further investigations, especially for the body index due to the variety of responses given by different authors, should be carried out.

DNA damage caused by microplastics has often been measured using the comet assay (single-cell gel electrophoresis), which measures DNA strand breaks in single cells [47]. This technique has been commonly used in human cells, but it has lately gained importance in the environmental and genetic toxicology of microplastics for different organisms [48], such as earthworms [49], mollusks [34,50,51], or fish [52]. It is not clear if microplastics alone can cause DNA damage, as some authors have opposite results when using this technique [34,53,54], and neither for microplastics with added pollutants [45,55]. Although most of the studies regarding DNA integrity and strand breaks perform the comet assay, it has certain limitations, as it cannot detect the small DNA fragments produced during apoptosis [47]. If microplastics cause cell death—not only single DNA strand breakages detected by the comet assay—then a test of general DNA degradation, such as those employed by Mičić et al. [38], could be used instead. This technique based on agarose gel can detect DNA degradation by the visualization of smears that can appear in the agarose gel indicating, depending on the brightness of the smear, the certain degree of degradation of the extracted DNA [56]. This technique has been previously used for studying DNA degradation on frozen beef [57] or for DNA degradation caused by oxidative damage [58].

The aim of the present study is to investigate physical and DNA integrity changes derived from polystyrene microplastics. For that purpose, the commercial species *Mytilus galloprovincialis* (Mediterranean mussel) was exposed to virgin polystyrene microparticles, polystyrene being one of the most common types of plastic found in the marine environment, at different concentrations during a medium time period (21 days). No hazardous compounds were added during the experiment in order to assess if microparticles can induce these changes without biomagnification of other compounds. Studies investigating the uptake of microplastic showed a high accumulation in gills [55,59,60], through microvilli activity and endocytosis [59]; thus, we expect that this organ will experience serious DNA damage.

## 2. Material and Methods

### 2.1. Experimental Design and Procedures

A total of 61 adult individuals of *M. galloprovincialis* were collected in January 2020 from El Puntal beach (43°31′33′′ N, 5°23′17′′ W), situated on the coast of Asturias (Spain). The individuals were immediately transported to the facilities of the Aquarium of Gijón (Asturias, Spain), where the experimental part was conducted. Mussels were allocated randomly in four independent 40 L tanks, with 16 mussels in the control and 15 mussels in each concentration analyzed, and allowed to acclimate for one week. Subsequently, each group was exposed daily to four different concentrations of polystyrene microparticles (size 10 µm, density 1.05 g/cm^3^) for two hours for 21 days.

Paul-Pont et al. [20] recommend to consider realistic ecosystem scenarios when designing experiments to assess the effects of exposure to microplastics on marine organisms. Exposure concentrations of microplastics chosen were realistic levels (in C1), similar to those we can find in the environment [20], and higher doses to which mussels could be further exposed (C2 and C3), in accordance with experiments performed by Lu et al. [61] in zebrafish (*Danio rerio*), and Wang et al. [35] in mussel (*M. curuscus*). Experimental treatments were control or C0 (no microplastics), group 1 or C1 (0.02 mg/L of microbeads), group 2 or C2 (0.2 mg/L), and group 3 or C3 (2 mg/L). In realistic conditions, intertidal mussels living in fluctuating environments are rarely exposed constantly to the same concentration of microplastics. Microplastics coming from the ocean or from adjacent rivers are carried by tidal movements and washed by waves, thus exposure is irregular and often recurrent. Thus, we have opted for an experiment of intermittent acute exposure (for a short time repeated over days). Mussels were daily transferred from the tanks to 5 L glass chambers where microbeads were added for two hours. The time of exposure was calculated based on a mussels’ filtration rate of 300 mL/min; with 15 mussels in the water volume of the experimental chambers, the totality of water was filtered in less than two hours. Mussels were then transferred back to the tanks.

Mussels were kept in an open circuit of tanks with filtered and aerated seawater. Marine phytoplankton gel in a mineral suspension was used to feed the mussels every two days, always after the exposure to microbeads, not before. After 21 days, mussels were transported, in the same glass chambers where the experiment was conducted in order to avoid stress, to the facilities of the University of Oviedo. Once in the laboratory, gills of each mussel were immediately excised to prevent possible DNA degradation due to cell death during manipulation of the specimens, and then were preserved in 1.5 mL Eppendorf tubes with ethanol for further analysis. After leaving them to settle, the precipitate found in the bottom of the tubes was taken and placed on a glass slide and visualized under the microscope for examination of microplastics in the gills. The rest of the body was employed to calculate body condition index.

### 2.2. Microbeads Employed

Polystyrene particles were chosen because they have a medium density, and therefore can be present not only in the water column, but also in sediments. Moreover, polystyrene particles can release toxic monomers and other chemicals used for their manufacture [24], and therefore the potential effects that can be caused in marine organisms can be greater than other polymers. In this experiment, we used microparticles based on polystyrene (C_8_H_8_)_n_, 10 µm size (std dev < 0.2 μm, coeff var < 2%), in aqueous suspension, 1.05 g/cm^3^ density, and 10% (solids) concentration (Sigma Aldrich, Germany, ref: 72986-10ML-F). Particle size used was 10 µm diameter, as smaller particles have the ability to translocate into the circulatory system in *M. edulis* [24], and this was beyond the scope of the present investigation.

### 2.3. Condition Index

Condition index (CI) is broadly used to measure the nutritional status of bivalves. In our study, the formula proposed by Baird et al. [62] was used:

(1) $$Condition\;index\;\left(CI\right)=\frac{Soft\;body\;wet\;weight\;}{Total\;weight}$$

If the calculated index has a value between 0.15 and 0.25, it indicates that the bivalve has a good nutritional status [44]. As gills were previously taken, calculated CIs were expected to be lower than the real values if all the organs were intact. Thus, results are valid for comparison between groups but should not be taken as absolute indicators of the physiological or nutritional status for each mussel.

### 2.4. DNA Extraction and Electrophoresis

First, ethanol-preserved gills were dried and DNA was extracted using an extraction kit designed for the recovery of genomic DNA from mollusks (E.Z.N.A.^®^ Mollusc DNA Kit) following manufacturer’s recommendations. In brief: samples were homogenized and lysed in a high salt buffer (CTAB) with 25 µL of proteinase k, incubated at 37 °C overnight, and extracted with chloroform to remove mucopolysaccharides. DNA purification was performed through several centrifugations with different buffers (ML buffer, BL buffer, HBC buffer (guanidinium chloride), ethanol-based DNA wash buffer, and elution buffer 10 mM Tris-HCl pH 8.5), in order to remove salts, proteins, and other contaminants. The DNA extracted was then quantified using a spectrophotometer Shimadzu UV1280 at 260 nm wavelength. For each sample, aliquots of 5 ng/µL were prepared and then, 10 µL (total mass of 50 ng) of each sample was charged into an 1.3% agarose gel and run at 80 mV for 2 h, as indicated in Mičić et al. [38] for the detection of apoptosis from mussel gill DNA. Molecular weight marker Perfect™ 100–1000 bp (EURx) was employed as ladder. Staining was performed with 2 µL of bromophenol blue sucrose solution and DNA was visualized on agarose gels under UV illumination NuGenius (Syngene) and photographed with a camera integrated in the same transilluminator.

**Figure 1:**
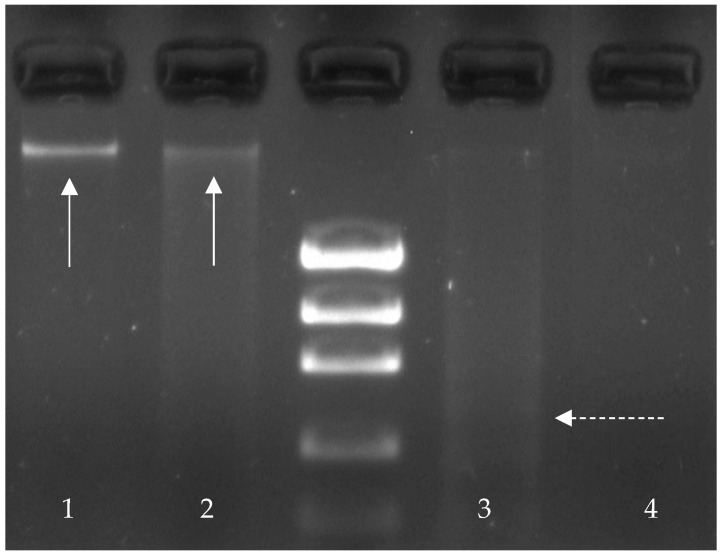
Samples scoring 1 to 4 for the different degrees of DNA degradation found in our study. Arrows shows genomic DNA, and broken arrow shows smear which indicates DNA degradation.

The DNA integrity was inferred from the migration pattern in the gel. Samples were classified into four different categories, depending on the level of DNA degradation, following criteria based on Quinet et al. [63]. When the genomic band is compact and completely defined with no smear or lighter bands, the DNA is considered not degraded, and therefore the sample is classified as 1. When the genomic band is almost inexistent and there is a high amount of smear, DNA is considered highly degraded, and it will be classified as 4. In between, when the genomic band is bright and the smear is light, the DNA has certain levels of degradation (category 2); when the genomic band is lighter or difficult to see and the smear has some bright, the DNA is quite degraded (category 3) (Figure 1). Apoptosis would be detected as a DNA ladder with clear, distinguishable bands. Three independent observers scored each sample, and the mean was used for the statistical analysis.

### 2.5. Data Analysis

The software used for conducting all the statistical analysis was PAST software [64]. For the statistical analysis of the condition indexes, after checking normality with Shapiro–Wilk test, ANOVA analysis was performed in order to determine if there is any significant difference between the different groups, followed by a Dunn’s multiple comparison test in order to resolve which specific means are significantly different from the others (Bonferroni corrected *p* values). For the DNA integrity, due to the non-normality of the data, a Kruskal–Wallis test was applied in order to determine if there were differences in DNA degradation between the four different groups, followed by post-hoc pairwise Mann–Whitney test to determine where the differences were.

## 3. Results

### 3.1. Mussels’ Status

No mortality was observed at the end of the experiment for any treatment, indicating that all of the effects of the experiment were at sub-lethal level. Acclimation was confirmed, as every mussel was attached to the bottom of the tanks after a week, and filter feeding was good, as every group had fecal pellets in the bottom of the tank. Microspheres were found in the precipitate of the gill samples treated, confirming that they were effectively adhered at the gill’s tissue.

### 3.2. Condition Index

**Figure 2:**
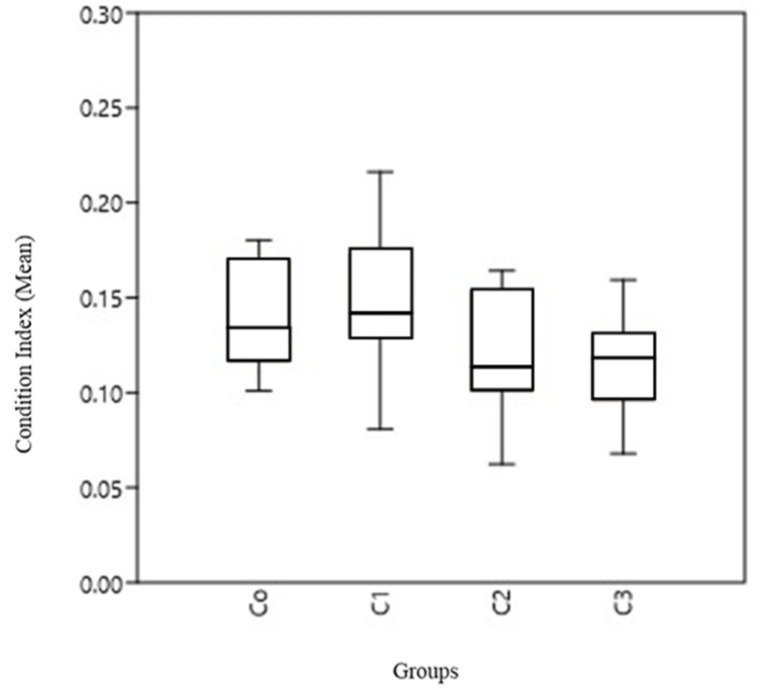
Boxplot for the condition indexes found in the different experimental groups.

As expected, the condition index values were slightly lower than 0.15, which is the minimum value for which the nutritional status is considered optimal. Raw data are presented in Supplementary Table S1. Means ranged from 0.146 (SD 0.034) in group 2 to 0.113 (SD 0.025) in group 3 (Figure 2).

**Figure 3:**
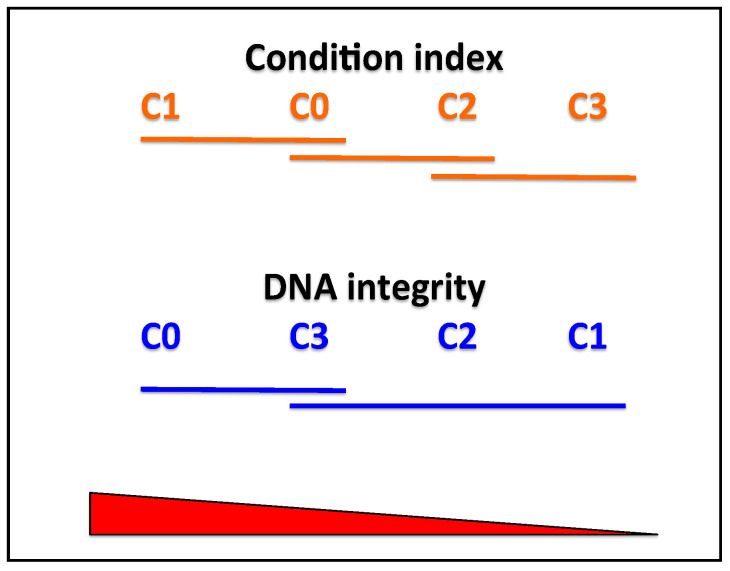
Graph showing the results of post-hoc tests for condition index (above) and DNA integrity (below) in the four experimental groups.

Statistical analysis showed a significant difference between the four different groups (*p* = 0.0039, df = 3, F = 4.96), and the Dunn’s post hoc (Table 1) showed significant differences between group C1 and groups C2 and C3 (*p* = 0.007 and *p* = 0.004, respectively), and between C0 and C3 (*p* = 0.033), the difference between C0 and C2 being only marginally significant (*p* = 0.053 < 0.10). Thus, as visualized in Figure 3, three overlapping groups appear regarding CI: one with the best condition containing C1 and C0, another intermediate with C0 and C2, and finally the group with the worst condition containing C2 and C3.

### 3.3. DNA Integrity

**Figure 4:**
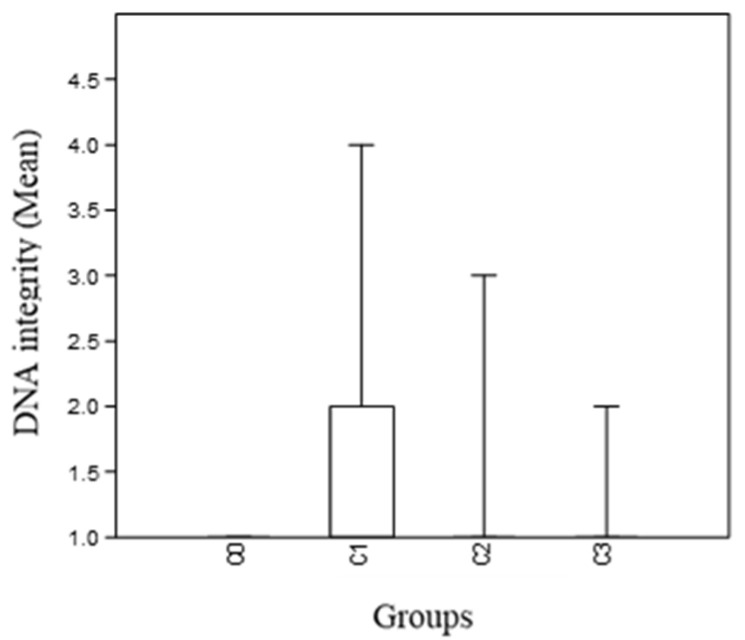
Boxplot for DNA integrity means in the different experimental groups.

High molecular weight DNA fragments (approximately 50–300 kpb) are seen as a band that migrates a short distance and can be interpreted as integer genomics DNA. Scores given to each individual by the three independent observers only differed in two of the 61 samples analyzed (Supplementary Table S1), for which the final score was the mean of the scores given by the three observers. DNA degradation in the C0 (control) group was inexistent (Supplementary Figure S1), as in every sample the genomic band was perfectly defined, and in group 3 only one of the samples had a mark different from 1. Regarding C1, five out of fifteen individuals had a value different from 1, showing a certain degree of degradation, and lastly, group 2 differed from 1 in three individuals (Supplementary Table S1, Figure 4).

The Kruskal–Wallis test performed showed a significant difference between sample medians (*p* = 0.026); and the Mann–Whitney test showed a significant difference between the control and group 1 (*p* = 0.10) and control and group 2 (*p* = 0.045) (Table 2). The post-hoc test exhibited a group containing C0 and C3, and another containing C3, C2, and C1.

**Figure 5:**
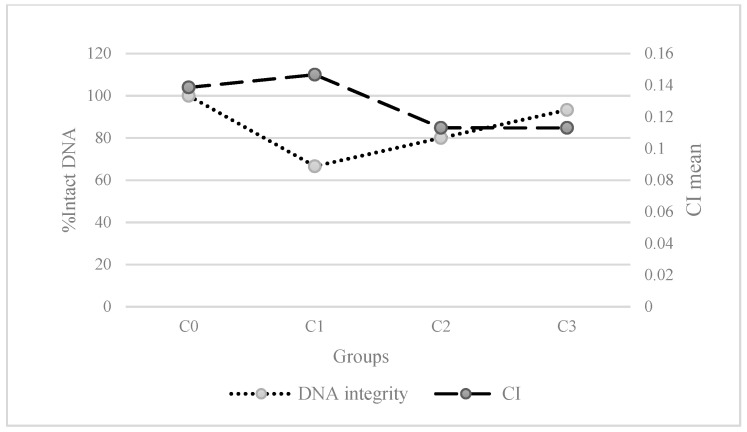
Diagram representing the trends of the different groups for condition index (CI), and the proportion of individuals with integer DNA (score = 1 for DNA integrity).

The summary of the post-hoc tests displayed in Figure 4 shows that C3 and the control group (C0) are grouped together with a higher DNA integrity (low or unperceived DNA degradation), while C2 and C1 were grouped together. In Figure 5, we can see clearly that the trends of DNA integrity (in number of individuals of category 1) and CI are opposite.

Clear signals of apoptosis, such as a ladder of multiple bands [38], were not detected with the method employed in this study. However, weak secondary light DNA bands were found for two treated individuals: individual 4 of C3, and individual 9 of C2 (Supplementary Figure S2). They might be early signals of apoptosis, but this cannot be ensured with this method.

## 4. Discussion

Microplastic contamination can have numerous impacts in marine organisms. In this study, significant effects on the DNA integrity and body condition in *M. galloprovincialis* have been shown after a medium-term exposure (21 days) to microplastics. The physical status of the individuals measured though the condition index showed a clearly suboptimal status for groups C2 and C3 (mean CI = 0.14 and 0.11, respectively). This could indicate that feeding behavior or the nutritional status is altered by microplastic ingestion at not too high concentration levels (0.2 and 2 mg/L). These can be due to their filtering-feeding behavior, as bivalves present efficient rates of microplastic ingestion [65] and microplastics can accumulate at cellular and subcellular levels, with a higher concentration in gills and digestive system [59]. In fact, in our study, microspheres were detected in gills, which concur with the aforementioned studies.

Moreover, microplastics can aggregate in digestive tissues and branchial epithelial cells [33], leading to a false satiated state, and decreasing fatty acid metabolism [65]. Therefore, the accumulation of microplastics in the digestive system may lead to a suboptimal health status, as shown in this study. Perhaps the effects cannot be generalized to all mussels; for example, Santana et al. [44] showed no physical impacts on the mussel *Perna perna* after 90 days of exposure to 0.1–1.0 μm PVC particles at 0.125 g/L. Studies reporting physical damage of microplastics on mussels normally use higher doses of microplastic concentration than the levels used in this study [34,44]; a novelty of our study is the confirmation that suboptimal conditions can be reached after a medium-term exposure to lower and realistic [20] levels of microplastics concentrations, especially for the lowest doses.

Differences between groups in our experiment could be explained as mussels adjusting their ingestion rates by increasing their filtration, but only up to a maximum, after which they experience a decrease in the filtration: a process called a unimodal response [66]. Mussels that have a high daily dose of microplastics may have reached the maximum filtration rate for a high number of particles floating in the tank, accumulating more microplastics in their stomach; or, conversely, they may have decreased their filtration rates, leading either way to this suboptimal status. The second explanation is consistent with the study performed by Woods et al. [29] in which mussels (*M. edulis*) were shown to decrease their filtration rates at higher levels of microplastic concentrations, although results for the mentioned study did not show differences in the condition index. Wegner et al. [67] showed that mussels (*M.* *edulis*) exposed to nanopolystyrene beads (100 nm) were able to detect these particles, thus reducing the opening of the valve, and therefore reducing filtration rates. These explanations all together may explain the differences found between groups; in the case of C1, the particle concentration may be too low for the animals to detect the microspheres, or simply the plastic accumulation is not enough for a false satiated status.

In addition to physical damages, microplastics can cause grave problems in the DNA of filter-feeding organisms. In the present study, the DNA integrity in gills showed significant differences between groups, especially between C1 and C2 (intermediate microbeads concentrations, with more individuals exhibiting DNA degradation in gills) and the control. These results suggest that the direct physical interaction of the gills with polystyrene microparticles has altered DNA in the gill cells, perhaps increasing cell mortality, since microplastics accumulate in gills [55,60]. The physical interaction with virgin microplastics seems to trigger DNA strand breaks in hemocytes in *M. galloprovincialis* [33], and our results would extend DNA damage beyond strand breaks to higher degrees of DNA degradation, although not at the highest concentration assayed. Contrasting results of CI and DNA degradation (Figure 5) suggest a trade-off between physical and DNA damage in mussel gills. If mussels exposed to highly concentrated microplastics (such as C3) close their valves and reduce filtration rates [29,67], they will shorten the time of exposure and thus the rate of DNA damage. Mussels exposed to lower microplastic concentrations may have normal filtration rates, increasing their direct exposure of the gills to microplastics, and therefore having higher DNA damage but a better physical condition (Figure 5). This would explain the absence of dose dependence of DNA damage in mussels found in other experiments with microbeads, where individuals exposed to higher microplastics concentrations had no significantly higher DNA damage [13,53].

Regarding the experimental design, the type of exposure employed in our study does not fit with the typical models of exposure of most studies (acute or chronical), since mussels were exposed to microplastics exposure every day for only two hours. It is known that mussels have rapid ingestion and egestion rates of microplastics when treated with acute exposure to high microplastics concentrations, showing inflammatory responses due to cleaning and recycling processes occurring during digestion [68]. Acute exposure, especially the higher doses, can represent punctual microplastics spillages, as it happens during periods of heavy storms, sewer overflow, and drops in the efficiency of wastewater treatment plants [69]. On the other hand, mussels have the ability to acclimate to a chronical long-term exposure [44]. This could be the case of species that are constantly submerged in polluted waters with little or no movement, nor wave wash. However, mussels living in the intertidal, such as *Mytilus galloprovincialis*, would not fit to either of these models. Exposing mussels to acute and daily exposition, mussels are forced into a daily depuration process, without having the opportunity to acclimate to microplastics, simulating environmental conditions of the intertidal. This type of exposure is consistent with the recommendation of Paul-Pont et al. [20] of considering realistic scenarios when designing experiments to assess the effects of exposure on marine organisms.

Overall, although significant differences were found in DNA degradation, clear signs of apoptosis were not found in our study. Virgin pellets do not add the effect of toxic compounds found in the environment that can be adsorbed by microplastics and transferred into the mussel’s tissue, magnifying their effects [70]. Avio et al. [33] showed that the differences between virgin pellets and contaminated pellets were not significant, independently of having pollutants adhered, which was the same conclusion as Pittura et al. [55]. Moreover, we used smooth microplastics, while microplastics with irregular surfaces or fibers can enhance the physical and DNA damage, as they can get entangled easily in the intestinal tract, prolonging retention rates, and therefore augmenting the time of damage. [14]. Genotoxic effects may vary greatly depending on the organism, the concentration and type of polymer, and even the shape [13]. Cole et al. [34] did not find DNA strand breaks in *M. edulis* after 7 days of exposure to different polymers sizes, and neither did Pittura et al. [55] after 28 days exposure to LPDE for *M. galloprovincialis*. In contrast, DNA damage has been reported for the same species in hemocytes exposed to virgin microplastics [33]: in the earthworm *Eisenia fetida* [49], in the clam *Scrobicularia plana* [50], and in fish larvae [52]. Thus, it seems that the DNA degradation signals found in our work (with other methods) would concur with those results. Lastly, not only has DNA damage been reported in marine organisms, but also genotoxic effects derived from an exposure to polystyrene particles has been recently reported in human cells [71], possibly extending the problem of microplastics in the marine environment to human consumers in the near future.

The implications of our results are important for conservation. This species covers a wide geographical range and is a bioindicator of marine coastal microplastic pollution [72]. Their direct exposure to microplastics, due to their intense filter-feeding activity, makes this species more vulnerable to this type of pollution [73]. The ever-increasing concentration of microplastics in marine environments is leading to a broad range of physical and toxicological effects on marine animals [13], and therefore the real risk that these hazardous compounds may suppose should continue being assessed.

## 5. Conclusions

This study confirmed physical and DNA damage of polystyrene particles at environmental doses after a medium-term exposure of *M. galloprovincialis*. Alterations in the condition indexes were greater in mussels exposed to higher doses of microparticles; however, DNA damage in gills was lower at these higher concentrations. This may be interpreted from the active recognition of microplastics by mussels making them in order to reduce filtration rates at higher concentrations, lowering the physical condition but protecting the gills from direct physical interaction with microplastics. Overall, DNA damage was low but not negligible. Further investigations are recommended with different environmental levels of microplastic concentrations in order to understand the potential impact of this emerging contaminant in *Mytilus* mussels.

## Figures and Tables

**Table 1:** Dunn’s post hoc for condition index (Bonferroni corrected *p* values). Significant differences shown in bold.

	Control	C1	C2	C3
Control		0.423	0.053	**0.033**
C1	0.426		**0.007**	**0.004**
C2	0.053	**0.007**		0.845
C3	**0.033**	**0.004**	0.845	

**Table 2:** Pairwise Mann–Whitney test results (*p* value above, U value below). Significant differences marked in bold.

	Control	C1	C2	C3
Control		**0.011**	**0.045**	0.178
C1	75		0.365	0.070
C2	90	95		0.292
C3	105	81.5	97	

## Data Availability

All data can be found in supplementary materials.

## Supplementary Materials

The following are available online at https://www.mdpi.com/article/10.3390/ani11082317/s1, Figure S1: All the samples loaded in an agarose gel. From left to right: individuals 1 to 15 (16 for the control group). From up to bottom: each line represents one group (C0 to C3). Figure S2: Image of the two samples found with sec-ondary light DNA bands (signalled with an arrow). Table S1: Condition indexes and DNA integrity scores for each individual.
