# Supplementary material for: Virgin Polystyrene Microparticles Exposure Leads to Changes in Gills DNA and Physical Condition in the Mediterranean Mussel Mytilus Galloprovincialis
Source: Animals (Basel). 2021 Aug 5;11(8):2317. doi: 10.3390/ani11082317 (PMC8388471; doi:10.3390/ani11082317)
Supplement: Supplementary file 1 [file animals-11-02317-s001.zip › animals-1314187-supplementary.pdf]

Supplementary Table S1: Condition indexes and DNA integrity scores for each individual.

Individual	Group	DNA integrity				Condition Index
		Observer 1	Observer 2	Observer 3	Mean	
1	Control	1	1	1	1	0.11
2	Control	1	1	1	1	0.16
3	Control	1	1	1	1	0.15
4	Control	1	1	1	1	0.12
5	Control	1	1	1	1	0.13
6	Control	1	1	1	1	0.16
7	Control	1	1	1	1	0.12
8	Control	1	1	1	1	0.11
9	Control	1	1	1	1	0.13
10	Control	1	1	1	1	0.14
11	Control	1	1	1	1	0.17
12	Control	1	1	1	1	0.18
13	Control	1	1	1	1	0.17
14	Control	1	1	1	1	0.10
15	Control	1	1	1	1	0.12
16	Control	1	1	1	1	0.18
1	Conc.1	1	1	1	1	0.20
2	Conc.1	4	4	4	4	0.18
3	Conc.1	1	1	1	1	0.14
4	Conc.1	1	1	1	1	0.17
5	Conc.1	1	1	1	1	0.14
6	Conc.1	1	1	1	1	0.13
7	Conc.1	1	1	1	1	0.08
8	Conc.1	2	2	2	2	0.13
9	Conc.1	3	4	3	3.3	0.22
10	Conc.1	1	1	1	1	0.15
11	Conc.1	1	1	1	1	0.12
12	Conc.1	4	4	4	4	0.13
13	Conc.1	1	1	2	1.3	0.13
14	Conc.1	1	1	1	1	0.17
15	Conc.1	1	1	1	1	0.16
1	Conc.2	1	1	1	1	0.16
2	Conc.2	1	1	1	1	0.10
3	Conc.2	1	1	1	1	0.13
4	Conc.2	3	3	3	3	0.15
5	Conc.2	1	1	1	1	0.06
6	Conc.2	1	1	1	1	0.13
7	Conc.2	1	1	1	1	0.08
8	Conc.2	1	1	1	1	0.10
9	Conc.2	1	1	1	1	0.11
10	Conc.2	1	1	1	1	0.07
11	Conc.2	1	1	1	1	0.16
12	Conc.2	2	2	2	2	0.11

13	Conc.2	2	2	2	2	0.12
14	Conc.2	1	1	1	1	0.16
15	Conc.2	1	1	1	1	0.11
1	Conc. 3	1	1	1	1	0.13
2	Conc. 3	1	1	1	1	0.16
3	Conc. 3	1	1	1	1	0.09
4	Conc. 3	2	2	2	2	0.07
5	Conc. 3	1	1	1	1	0.10
6	Conc. 3	1	1	1	1	0.15
7	Conc. 3	1	1	1	1	0.12
8	Conc. 3	1	1	1	1	0.13
9	Conc. 3	1	1	1	1	0.11
10	Conc. 3	1	1	1	1	0.12
11	Conc. 3	1	1	1	1	0.13
12	Conc. 3	1	1	1	1	0.15
13	Conc. 3	1	1	1	1	0.08
14	Conc. 3	1	1	1	1	0.10
15	Conc. 3	1	1	1	1	0.12

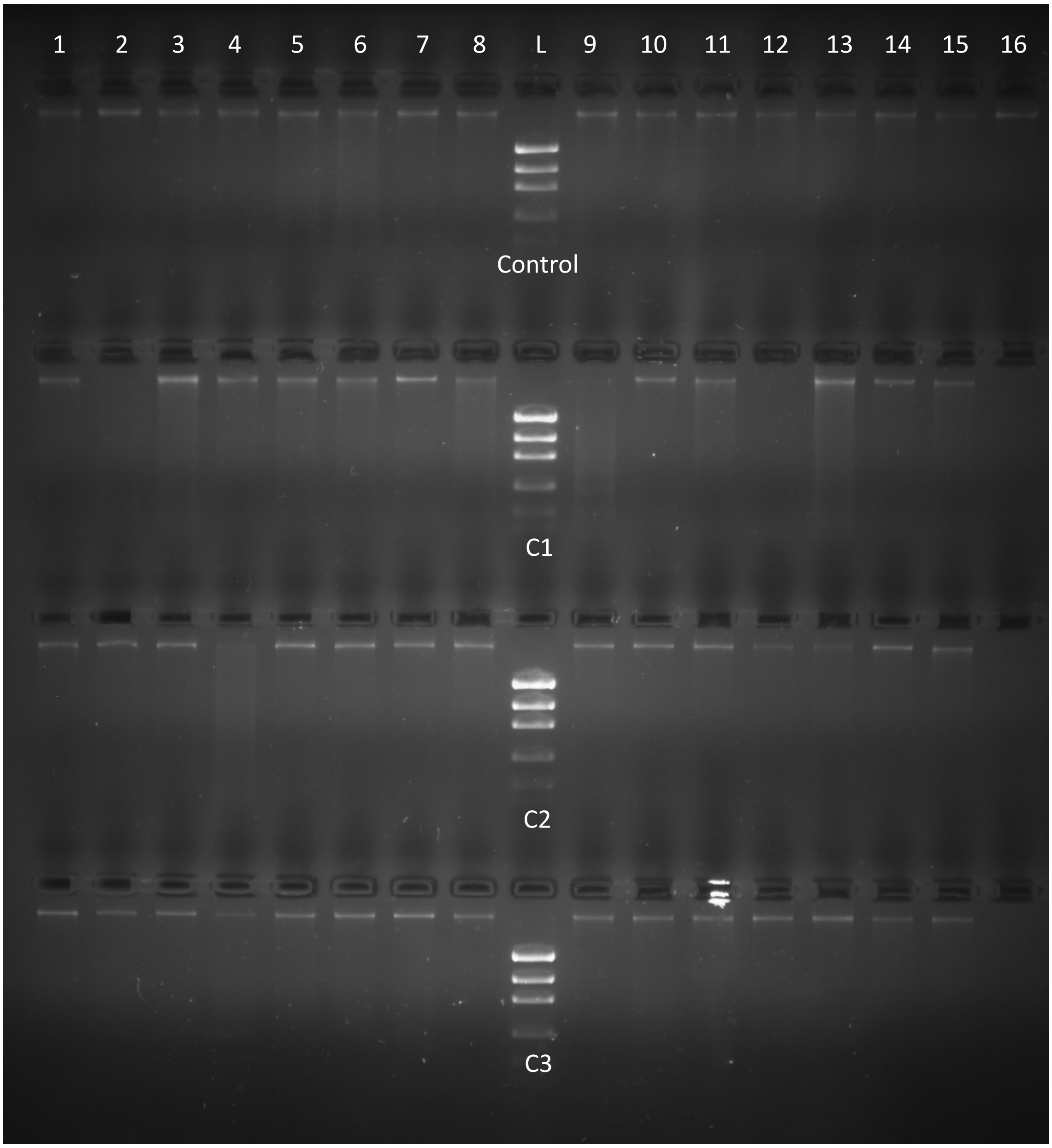

Supplementary Figure S1: All the samples loaded in an agarose gel. From left to right: individuals 1 to 15 (16 for the control group). From up to bottom: each line represents one group (C0 to C3)

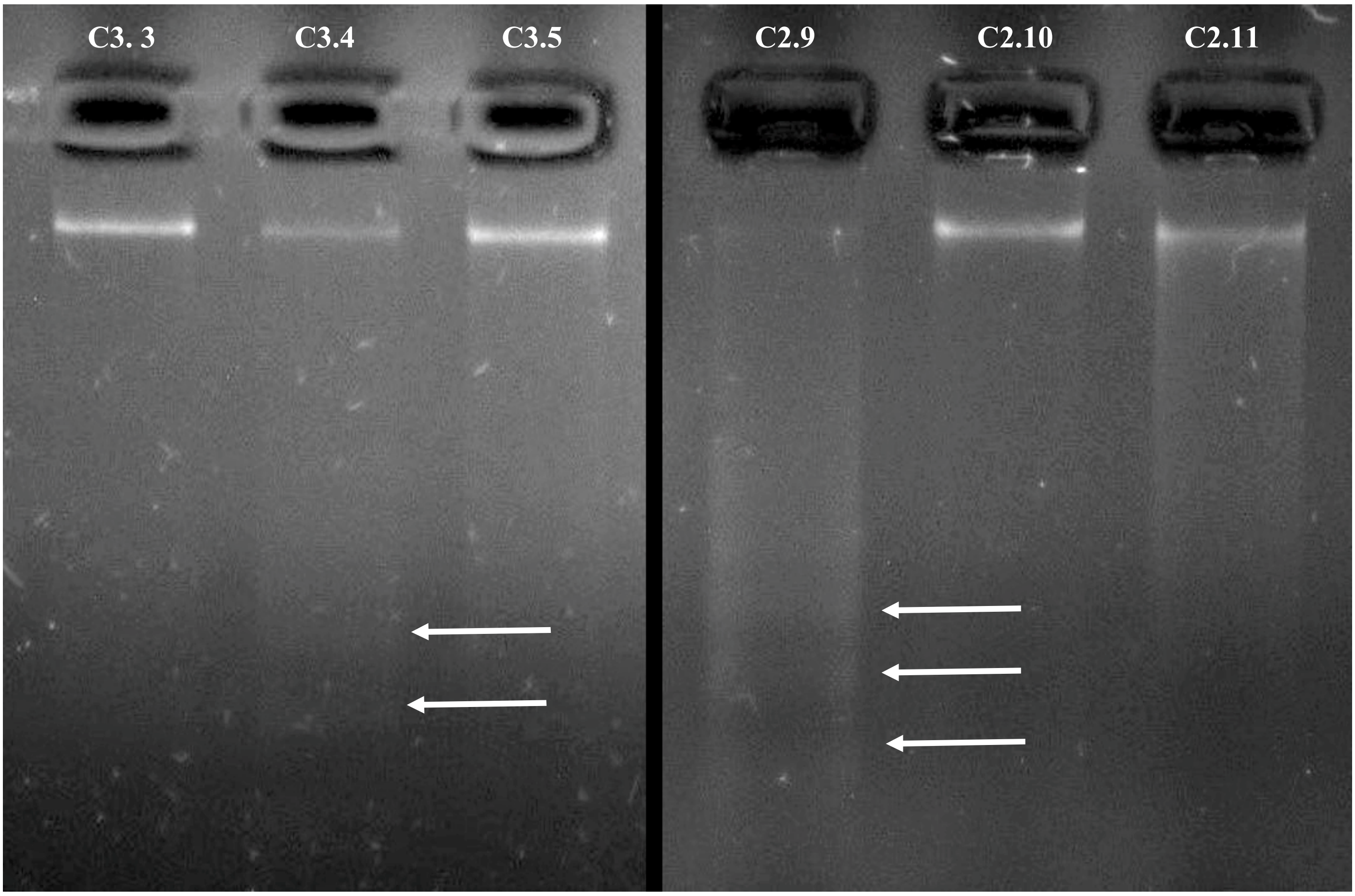

Supplementary Figure S2: Image of the two samples found with secondary light DNA bands (signalled with an arrow).
